# Phenotypic and Functional Characterizations of Mesenchymal Stem/Stromal Cells Isolated From Human Cranial Bone Marrow

**DOI:** 10.3389/fnins.2022.909256

**Published:** 2022-06-07

**Authors:** Kaichuang Yang, Ruijie Lu, Jianan Lu, Shucai Fan, Qiang Zhang, Zijian Lou, Yuyuan Ma, Gang Lu, Ruolang Pan, Jianmin Zhang

**Affiliations:** ^1^Department of Neurosurgery, Second Affiliated Hospital of Zhejiang University, School of Medicine, Hangzhou, China; ^2^Center for Rehabilitation Medicine, Department of Neurosurgery, Zhejiang Provincial People's Hospital, Affiliated People's Hospital, Hangzhou Medical College, Hangzhou, China; ^3^The Second Clinical Medical College, Wenzhou Medical University, Wenzhou, China; ^4^Key Laboratory of Cell-Based Drug and Applied Technology Development in Zhejiang Province, Hangzhou, China; ^5^Institute for Cell-Based Drug Development of Zhejiang Province, S-Evans Biosciences, Hangzhou, China

**Keywords:** cranial bones, mesenchymal stem/stromal cells, transdifferentiation, immunomodulation, neural-like cells

## Abstract

Mesenchymal stem/stromal cells (MSCs) are adult stem cells that were originally isolated from bone marrow. In contrast to long bone-derived MSCs that have been extensively characterized, our knowledge regarding to MSCs isolated from flat bones (e.g., cranial bones) remain less clear. In this study, MSCs were purified from human cranial bone marrow (CB-MSCs) and their transdifferentiation capacity and immunomodulatory functions were further characterized. Phenotypic analysis of CB-MSCs demonstrated high expression of CD73, CD90, and CD105 while negative for CD14, CD34, and HLA-DR. Further *in vitro* differentiation assay shown that CB-MSCs capable of differentiating into cell types of mesenchymal origin (i.e., adipocytes, osetoblasts, and chondrocytes) and collectively, these results indicated that cells isolated from cranial bone marrow in this study are bona fide MSCs according to the minimal criteria proposed by the International Society for Cellular Therapy. Following *in vitro* expansion, single colony-derived CB-MSCs (scCB-MSCs) were obtained and confocal microscopy analysis further revealed functional heterogeneity within primary CB-MSCs. Specifically, obtained scCB-MSCs exhibited GABA progenitor features, as determined by olig2 and nestin. As expect, scCB-MSCs were readily induced to differentiate into GABAergic neuron-like cells. Furthermore, immunomodulatory roles of scCB-MSCs were evaluated following co-culture with human peripheral blood lymphocytes and results shown that co-culturing with scCB-MSCs significantly suppressed lymphocyte proliferation and promoted differentiation of lymphocytes into regulatory T cells but not Th1/Th17 phenotype. Overall, our results indicated that CB-MSCs exhibited clonal heterogeneity with marked propensity to differentiate into neural-like cells and this might represent promising candidates for the treatment of neurodegenerative diseases.

## Introduction

Mesenchymal stem/stromal cells (MSCs) are adult stem cells that are present and accessible from a range of human tissues, such as umbilical blood cord, adipose tissue, and bone marrow (Berebichez-Fridman and Montero-Olvera, 2018). Similar to stem cells of different origins, MSCs exhibited self-renewal capacity and remarkable differentiation plasticity. Following isolation, MSCs can be further manipulated *in vitro* to differentiate into multilineages with extensive expansion potential (Le Blanc and Davies, 2018). In addition to cell types of mesenchymal origin (e.g., adipocytes, osteoblasts, and chondrocytes), an increasing number of studies have demonstrated neurogenic potentials of MSCs under specific conditions (Jang et al., 2010; Yan et al., 2013). This transdifferentiation plasticity renders MSCs promising candidates to potentially restore damaged neurons and would possibly be a valuable source of stem cells for the treatment of neurodegenerative disorders (Volkman and Offen, 2017).

Given the vast variety of source tissues, isolated MSCs exhibited tissue-to-tissue and intra-population heterogeneity with marked propensity to differentiate into different lineages (Rebelatto et al., 2008; Warrier et al., 2012; Kwon et al., 2016). For example, umbilical blood tissue-derived MSCs differentiated into muscle cells more robustly in comparison to MSCs isolated from umbilical blood cord (Mishra et al., 2020). This further highlights the importance of selecting the most appropriate MSCs source for specific diseases. In the context of neurodegenerative diseases, MSCs derived from a variety of tissues, such as dental pulp, adipose tissue, bone marrow and umbilical cord, have been reported to exhibit neuronal differentiation potential (Jeong et al., 2004; Ullah et al., 2016; Urrutia et al., 2019). Among all tissues, cranial bone marrow have attracted interest as a promising source of MSCs with remarkable neurogenic potential. It has been reported that intravenous transplantation of MSCs isolated from cranial bone marrow (CB-MSCs) promoted functional recovery and improved motor function in rats after ischemic stroke and spinal cord injury, respectively (Abiko et al., 2018; Maeda et al., 2021). In line with these observations, previous studies in our lab demonstrated that human CB-MSCs were highly inducible into neuron-like cells with robust expression of neural markers (Ma et al., 2019). Morphology changes of CB-MSCs following *in vitro* neural induction was also observed using scanning electron microscopy (Ma et al., 2021). Collectively, these results indicate that CB-MSCs hold great potential as an alternative source of MSCs for the treatment of neurodegenerative diseases.

In this study, we reported on the *in vitro* cloning of CB-MSCs and further characterized transdifferentiation capacity and immunoregulatory functions of single colony-derived CB-MSCs (scCB-MSCs) in comparison to umbilical cord-derived MSCs (UC-MSCs). Our results demonstrated that isolated CB-MSCs exhibited marked transdifferentiation capacity and functional heterogeneity as scCB-MSCs displaying greater neurogenic potential. Further, scCB-MSCs have immunomodulatory functions that impact lymphocyte proliferation and differentiation. Overall, this detailed investigation further our understanding of differentiation plasticity and immunoregulatory roles of CB-MSCs and will be a stepping-stone for the development of MSCs-based therapies to treat neurodegenerative disorders and post-traumatic diseases.

## Materials and Methods

### *In vitro* Culture of CB-MSCs and Establishment of scCB-MSCs

CB-MSCs and scCB-MSCs were obtained using previously reported protocols with minor modifications (Jeon et al., 2011; Ma et al., 2019). Briefly, the cranial bones from the clinic were excised into chips of ~4–8 mm^3^, from which mononuclear cell content was flushed out using αMEM (Cellgro). Collected mononuclear cells (~1 × 10^6^) were seeded in a 10 cm tissue culture-treated petri dish (Sigma-Aldrich) and grown at 37°C, 5% CO_2_ and 95% humidity in αMEM supplemented with 10% FBS (Gibco), 2 mM L-glutamine (Sigma-Aldrich), 100 U/mL penicillin (Sigma-Aldrich), and 100 μg/mL streptomycin (Sigma-Aldrich). After 48 h incubation, non-adherent cells were completely removed by thoroughly changing the medium and remaining adherent cells were continuously cultured.

To establish scCB-MSCs, mononuclear cell content from cranial bones was seeded in first 10 cm dish for 6 h. The supernatant was transferred to a new dish and incubated for another 6 h. Non-adherent cells were then transferred to a new dish and incubated for 36 h. Finally, adherent cells in the three dishes were cultured in αMEM supplemented with 10% FBS for 14 d, and well-separated cell colonies appeared. Individual colonies were identified under phase-contrast microscope and enclosed using sterile cloning cylinders (10 mm in diameter; Sigma-Aldrich). Of note, selected colonies were well apart from each other to avoid multiple colonies within single cloning cylinder. After removing culturing medium within cloning cylinders, 50 μL of 0.25% trypsin (Gibco) was added to harvest and transferred scCB-MSCs (~100 cells) into a 48-well tissue culture plate (Sigma-Aldrich) for further culturing and expansion under the same condition detailed above.

### Phenotypic Analysis of CB-MSCs Using Flow Cytometry

Phenotype of isolated CB-MSCs was examined as previous study (Ma et al., 2019). Cells at passage of 5 were harvested using 0.25% trypsin and after three washes in phosphate-buffered saline (PBS), CB-MSCs were equally aliquoted (~1 × 10^5^ cells) into 1.5 mL Eppendorf tubes for staining. Specifically, CB-MSCs were centrifuged at 200 × g for 2 min and cell pellets were gently disrupted following the addition of 0.1 μg fluorescein isothiocyanate (FITC)/phycoerythrin (PE)-conjugated primary antibodies targeting HLA-DR (1:100; Abcam), CD14 (1:100; Abcam), CD34 (1:100; Abcam), CD73 (1:100; Abcam), CD90 (1:100; Abcam), and CD105 (1:100; Abcam), respectively. As controls, FITC/PE-conjugated mouse isotype IgGs (Thermo Fisher Scientific) were also included. All primary antibodies and isotype IgG were diluted in flow cytometry buffer (PBS containing 0.5% bovine serum albumin, 2 mM EDTA, and 0.05% NaN3). After 30 min incubation on ice, cells were washed in 1 mL of flow cytometry buffer and centrifuged at 200 × g for 2 min. Cell pellets were re-suspended in 100 μL of flow cytometry buffer and analyzed for surface staining using a NovoCyte flow cytometer (ACEA Biosciences). For each sample, 10,000 events were collected and all data were analyzed using NovoExpress® software (Agilent Technologies Inc).

### Examination of Multilineage Differentiation Potential of CB-MSCs

To further characterize the differentiation potential of isolated CB-MSCs, cells at passage of 5 were selected for following treatments as previously described (Ma et al., 2019). Briefly, cells were allowed to grow to ~100% confluency in tissue culture plates before replacing regular culture medium with NH OsteoDiff medium (Miltenyi Biotec) and NH AdipoDiff medium (Miltenyi Biotec) to induce the differentiation into adipocytes and osteoblasts, respectively. To induce chondrogenic differentiation, ~2 × 10^6^ cells were harvested and cultured in NH ChodroDiff medium (Miltenyi Biotec) in 15 mL Falcon tubes. All induction experiments were performed according to the manufacturer's instructions with each medium replaced twice a week. After *in vitro* induction, cells in tissue culture plates were stained using Alizarin Red (Sigma-Aldrich) and Oil Red O (Sigma-Aldrich) to examine osteogenic and adipogenic differentiation, respectively. For cells cultured in 15 mL Falcon tubes, cells were processed as previously reported in our lab (Ma et al., 2019), and finally stained with Alcian Blue (Sigma-Aldrich) to examine chondrogenic differentiation. All samples were imaged using phase contrast microphotography.

### Immunofluorescence Staining

CB-MSCs and scCB-MSCs were seeded onto coverslips in a 24-well tissue culture plate and allowed to adhere overnight. In the day of experiment, cells were washed in PBS three times and fixed in 4% paraformaldehyde (PFA; Sigma-Aldrich) prior to permeabilization using 0.5% Triton X-100 (Sigma-Aldrich) diluted in PBS. After 10 min permeabilization, cells were then blocked in 5% bovine serum albumin (BSA; Sangon Biotech) diluted in PBS for 10 min and then simultaneously stained for Nestin and Olig2 molecules using Alexa Fluor 647-conjugated anti-Nestin (1:100; Thermo Fisher Scientific) and FITC-conjugated anti-Olig2 antibodies (1:100; Thermo Fisher Scientific) diluted in antibody staining buffer (ABS; 0.05% sodium azide and 1% BSA in PBS). After 30 min staining at room temperature, cells were washed three times using PBS and finally, coverslips were mounted onto microscope slides containing a droplet of ProLong™ Gold Antifade Mountant with DAPI (Thermo Fisher Scientific) and allowed to stain nuclei and cure overnight at room temperature.

In some assays, scCB-MSCs and CB-MSCs on coverslips were first subjected to *in vitro* induction. Specifically, cells on coverslips were incubated in neuronal induction medium I composed of Ham's DMEM/F12 (Gibco) that supplemented with 2% FBS, 0.25 × B-27 (Thermo Fisher Scientific), 1 × N-2 (Thermo Fisher Scientific), 20 mM retinoic acid (Sig-ma-Aldrich) and 12.5 ng/ml basic fibroblast growth factor (bFGF; Thermo Fisher Scientific). After 7 days incubation at 37°C and 5% CO2, this neuronal induction medium I was replaced with neuronal induction medium II composed of DMEM/F12 (Gibco) that supplemented with N-2/B-27 and 300 ng/mL sonic hedgehod (Thermo Fisher Scientific) for an additional 7 days. Both neuronal induction media were changed every 2 days and at the day of 14, cells were processed as detailed above with the exception that gamma-aminobutyric acid (GABA) and neuron-specific class III beta-tubulin (TUJ-1) molecules were stained using APC-conjugated anti-GABA (1:100; Abcam) and Alexa Flu-or488-conjugated anti-TUJ-1 antibodies (1:100; Thermo Fisher Scientific). All samples were imaged using a FV1000 confocal laser scanning microscope (Olympus).

### Flow Cytometric Analysis of PBMCs Proliferation and Differentiation Upon Co-culture With scCB-MSCs

Peripheral blood samples were collected from healthy volunteers at Zhejiang people's hospital for isolating PBMCs as previously reported with minor modifications (Repnik et al., 2003). Briefly, 2 mL of blood samples were layered on 8 mL of Ficoll (Invitrogen) and after 400 × g centrifugation for 30 min, the buffy coat layer was subjected to ACK lysing buffer (Thermo Fisher Scientific) to remove red blood cells and washed in RPMI-1640 medium (Gibco) three times prior to experiments.

UC-MSCs which have been reported to regulate lymphocyte proliferation and differentiation were included here as a positive control and isolated as previously reported with minor modifications (Zhou et al., 2011; Chao et al., 2014; Chen et al., 2015). Briefly, an umbilical cord was cut into pieces (~2 cm in length) followed by a longitudinal cut to expose the blood vessels and surrounding Wharton's jelly; carefully scraped Wharton's jelly away from blood vessels and collected tissues were centrifuged at 400 × g for 3 min. Approximately 1 g of tissues were spread on the bottom of a T-75 flask using a cell scraper for primary adherent culture.

To evaluate the ability of scCB-MSCs to regulate PBMCs proliferation, isolated PBMCs were fluorescently labeled using CFSE (Thermo Fisher Scientific) and then ~5 × 10^5^ cells were plated on the bottom of transwell inserts (pore size 0.4 μm; Sigma-Aldrich) in RPMI-1640 supplemented with 10% FBS and 100 U/mL penicillin/streptomycin. After overnight incubation, transwell inserts were transferred into a tissue culture plate and co-cultured with UC-MSCs (~5 × 10^6^ cells) or scCB-MSCs (~5 × 10^6^ cells) prior to the addition of phytohemagglutinin (PHA; Sigma-Aldrich) at the final concentration of 10 μg/mL; transwell inserts alone stimulated with PHA but without co-culture were also included as a control. After 4 days of treatment, PBMCs and supernatants were collected to analyze proliferation rate and interferon α (IFN-α) levels using flow cytometry and enzyme-linked immunosorbent assay (ELISA) kit (Abcam), respectively.

To further examine the impact of scCB-MSCs on PBMCs differentiation, a similar experimental setup detailed above using transwell-based co-culture system was used with the exception that PBMCs were not fluorescently labeled with CFSE. After 4 days of co-culture, PBMCs were harvested from transwell inserts and aliquoted into two Eppendorf tubes for further analyses. Specifically, PBMCs were first incubated in Human TruStain FcX (Biolegend) for 30 min to block Fc receptors. PBMCs were then washed in PBS and incubated with PE/PerCP-conjugated antibodies (1:100; Thermo Fisher Scientific) targeting CD3 and CD8 molecules. After 30 min incubation on ice, PBMCs were washed in PBS and permeabilized using 0.5% Triton X-100 for 10 min prior to intracellularly stain IFN-γ and IL-17 using FITC/APC-conjugated antibodies (1:100; Santa Cruz; Thermo Fisher Scientific) for additional 30 min incubation on ice. To determine the effect of MSCson regulatory T cells (Treg) proliferation, PBMCs were co-cultured with UC-MSCs and scCB-MSCs without PHA treatment. Then the PBMCs were pre-blocked as mentioned above and stained for CD4, CD25 and CD127 using FITC/PE/APC-conjugated antibodies (1:100; Thermo Fisher Scientific) for 30 min on ice. After staining, all samples were washed in PBS and fixed in 4% PFA prior to flow cytometry analysis as detailed above.

### Statistical Analysis

Data were reported throughout as mean ± standard deviation (SD) and analyzed using GraphPad Prism 6.0 software (GraphPad Software). Statistical differences between the two groups were evaluated by Student's *t*-tests. *P* values of <0.05 were considered as statistically significant in this study.

## Results

### Phenotypic and Functional Characterization of CB-MSCs and Single Colony-Derived CB-MSCs (scCB-MSCs)

**Figure 1:**
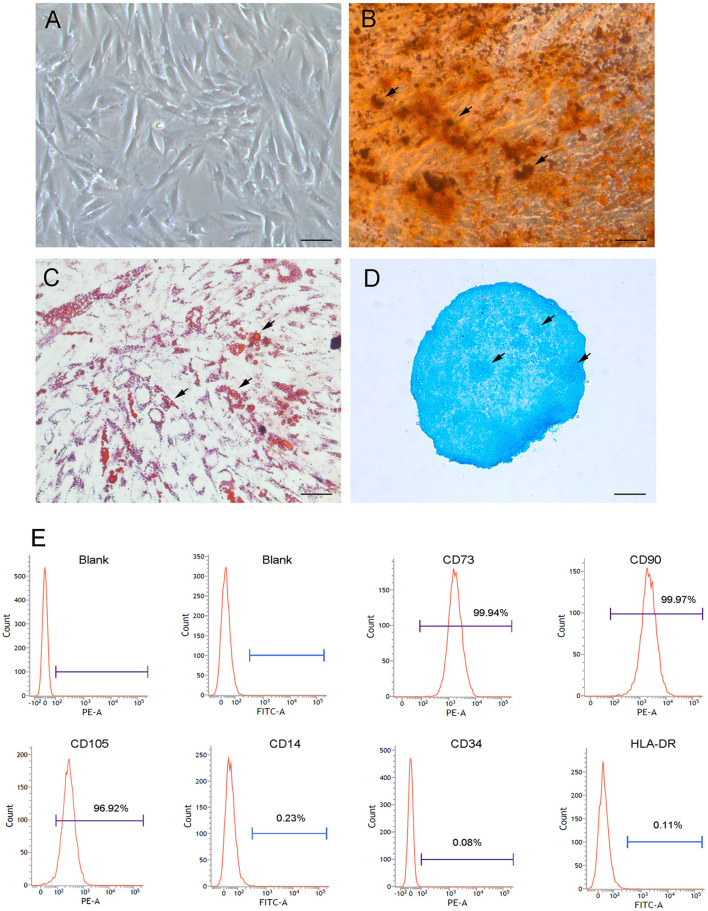
Morphological and phenotypic characterizations of CB-MSCs. **(A)** Morphology of cranial bone marrow-derived MSCs following *in vitro* culture at passage of 5 was imaged using phase contrast microphotography. Arrows showed mineral deposition **(B)** Adipogenic **(C)** and chondrogenic **(D)** differentiation after 3 weeks of *in vitro* differentiation using Alizarin Red, Oil Red O and Alcian Blue staining, respectively. Scar bar = 50 μm **(E)** Phenotypic analysis of surface markers using flow cytometry. MSCs were stained with FITC/PE-conjugated primary antibodies targeting selected surface markers (i.e., CD73, CD90, CD105, CD14, CD34, and HLA-DR). As controls, MSCs were also stained with fluorochrome-conjugated isotype IgGs to identify positive cells.

To characterize CB-MSCs, cells were first isolated and following *in vitro* culture, CB-MSCs exhibited a typical fibroblastic morphology similar to that of MSCs as spindle-shaped cells firmly attached to the plate (Figure 1A). *In vitro* differentiation assays shown that isolated CB-MSCs capable of differentiating into osteoblasts, adipocytes and chondrocytes in response to specific stimulus (Figures 1B–D). Real-time quantitative polymerase chain reaction (qPCR) for *Bglap, Leptin*, and *Collagen II* were further performed to validate the differentiation into mesenchymal derivatives (Supplementary Figure 1). Moreover, cell surface markers were analyzed using flow cytometry phenotyping according to the recommendations in the international society for Cellular Therapy (ISCT) guidelines (Dominici et al., 2006). The results showed that majority of CB-MSCs (>97%) were CD73+CD90+CD105+ and CD14^−^CD34^−^HLA-DR^−^ (Figure 1E).

**Figure 2:**
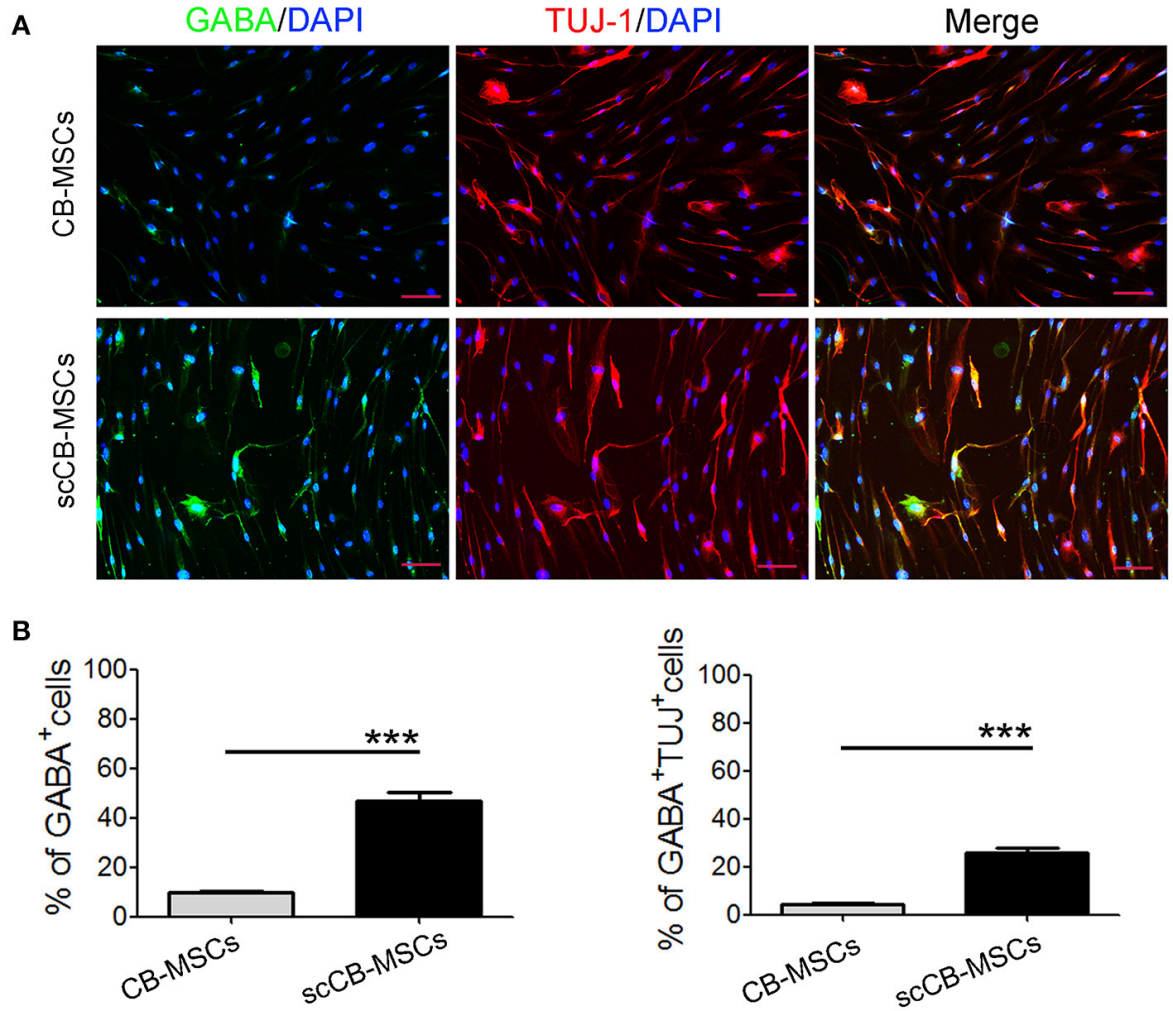
Confocal microscopic analysis of Nestin and Olig2 expression in CB-MSCs and scCB-MSCs. **(A)** CB-MSCs (top panel) and scCB-MSCs (bottom panel) were seeded on coverslips and permeabilized using 0.5% Triton X-100 for 10 min before the addition of Alexa Fluor 647/FITC-conjugated antibodies to stain Nestin (red) and Olig2 (green) molecules, respectively. After three washes in PBS, cells were further stained using DAPI to visualize nuclei (blue). Imaging was performed using a laser-scanning confocal microscope to examine the expression of Nestin and olig2. Scale Bars = 50 μm. **(B)** Percentages of positive cells were quantified and represented as mean ± SD of three independent experiments. Asterisks represent significant difference (^***^P < 0.01) compared to the control group.

**Figure 3:**
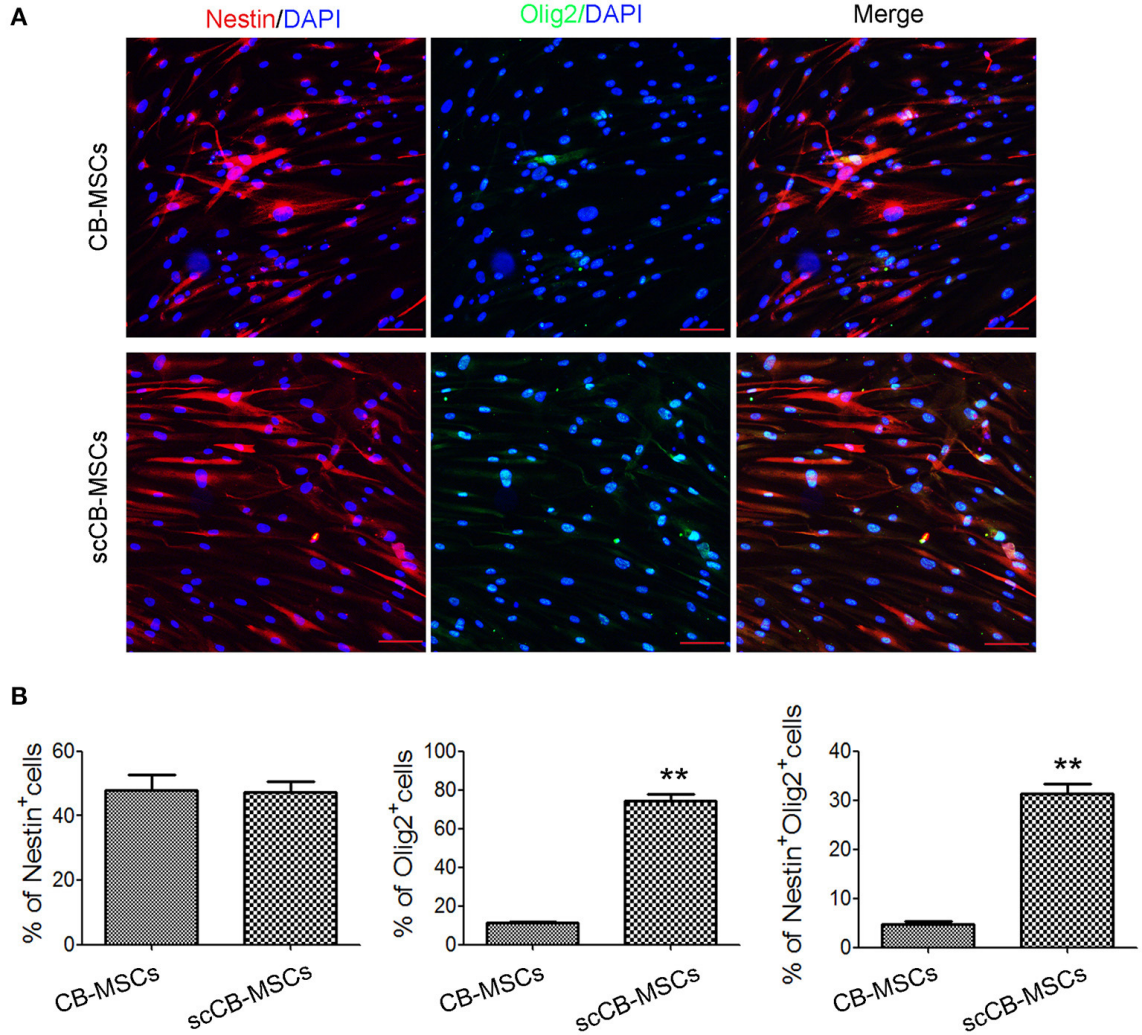
Induced neuronal differentiation of CB-MSCs and scCB-MSCs. **(A)** CB-MSCs (top panel) and scCB-MSCs (bottom panel) were seeded on coverslips and cultured overnight in regular MSC medium. In the next day, regular MSC medium was replaced with neuronal induction medium and after 2 weeks of differentiation, MSCs were washed in PBS and then permeabilized using 0.5% Triton X-100 before the addition of APC/Alexa Fluoa 488-conjugated antibodies to stain GABA (green) and TUJ-1 (red) molecules, respectively. After three washes in PBS, cells were further stained using DAPI to visualize nuclei (blue) before mounted onto microscope slides. Imaging was performed using a laser-scanning confocal microscope and percentages of MSCs that are positive for GABA and GABA/TUJ-1, respectively, were further summarized in **(B)**. Error bars represent mean ± SD of three independent experiments. Asterisks represent significant difference (^**^P < 0.001) compared to the control group. Scale Bars = 50 μm.

To evaluate the heterogeneity of isolated CB-MSCs, CB-MSCs were further cloned and scCB-MSCs were analyzed in comparison to CB-MSCs. Indeed, confocal microscopy revealed that although ~50% of both CB-MSCs and scCB-MSCs expressed Nestin, the percentage of cells expressed Olig2 was significantly higher in scCB-MSCs (~70%) than observed in CB-MSCs (~10%); as a result, nearly a third of scCB-MSCs co-expressed Nestin and Olig2 as opposed to only ~5% CB-MSCs exhibiting this phenotype (Figure 2). mRNA expressions of *Nestin* and *Olig2* determined by real-time qPCR further supported the finding in Immunofluorescence staining (Supplementary Figure 2). Further analysis showed that scCB-MSCs were highly inducible to transdifferentiate into GABAergic neuron-like cells as significantly higher percentage of GABA+ and GABA+TUJ−1+ cells were observed in scCB-MSCs compared to CB-MSCs under the same induction condition (Figure 3).

### Examination of Immunoregulatory Roles of scCB-MSCs on Peripheral Blood Mononuclear Cells (PBMCs) Proliferation and Differentiation

**Figure 4:**
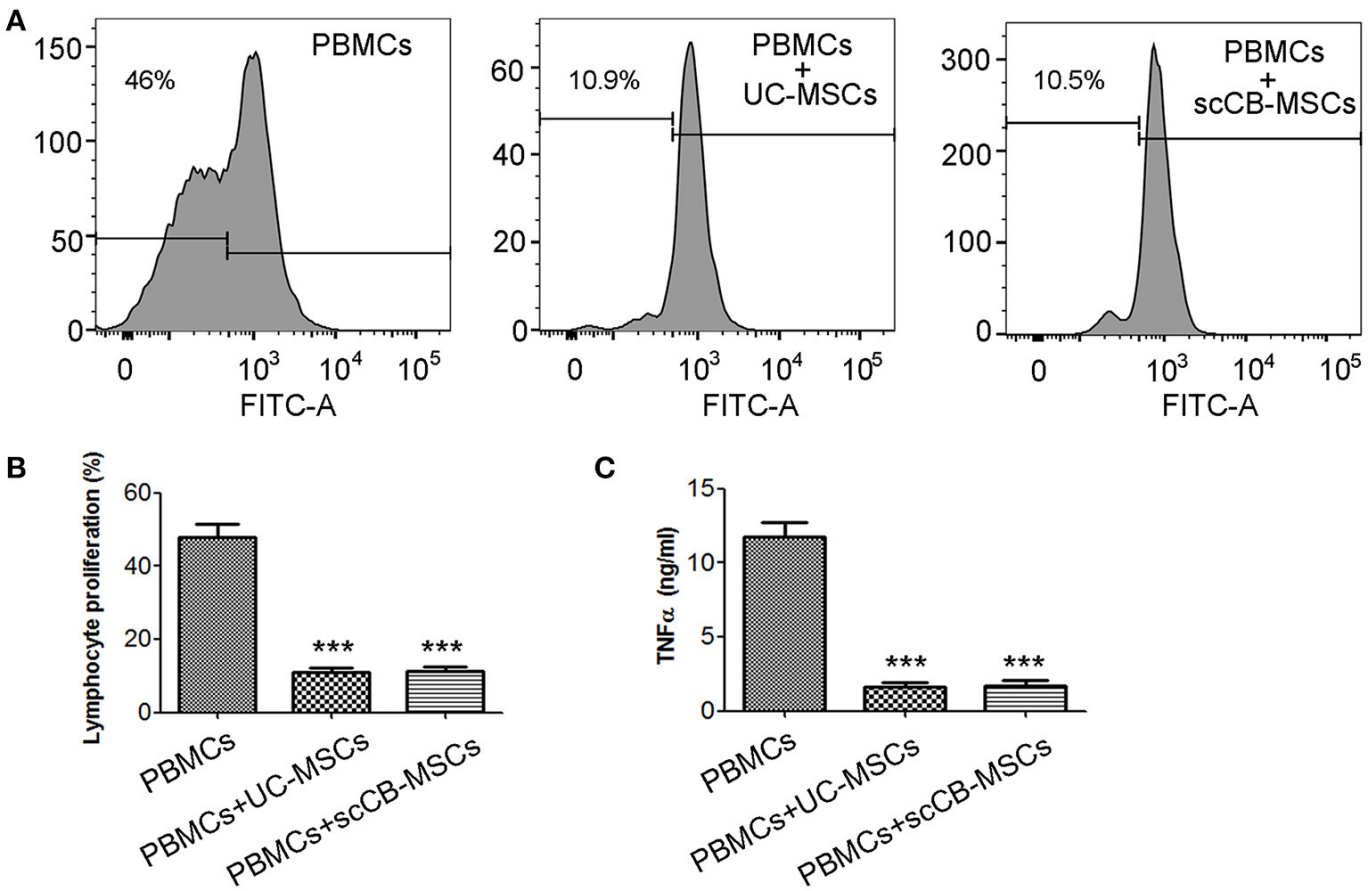
Inhibitory effects of scCB-MSCs on the proliferation of PBMCs. **(A)** Human PBMCs were labeled with CFSE and then CFSE-labeled PBMCs alone (left histogram), co-cultured with UC-MSCs (middle histogram) or scCB-MSCs (right histogram) at a PBMCs-to-MSCs ratio of 1:10, were further assessed on their ability to proliferate in response to polyclonal T-cell mitogen PHA stimulation. After 4 days, cells and supernatants were collected; cell proliferation was analyzed using flow cytometry and percentage of proliferating cells was summarized in **(B)** and TNF-α secretion levels in supernatants were also determined using ELISA and summarized in **(C)**. Error bars represent mean ± SD of three independent experiments. Asterisks represent significant difference (^***^P < 0.001) compared to the PBMCs alone group.

To investigate the impact of scCB-MSCs on proliferation of PBMCs, carboxyfluorescein diacetate succinimidyl ester (CFSE)-based proliferation assay was used. As shown in Figures 4A,B, ~50% PBMCs divided following PHA stimulation; this number is significantly reduced to ~10% in PBMCs co-cultured with umbilical cord-derived MSCs (UC-MSCs) and scCB-MSCs, respectively. Further analysis on cytokine secretion levels revealed a similar trend that co-culturing with UC-MSCs or scCB-MSCs significantly inhibited TNF-α secretions in PBMCs (Figure 4C).

**Figure 5:**
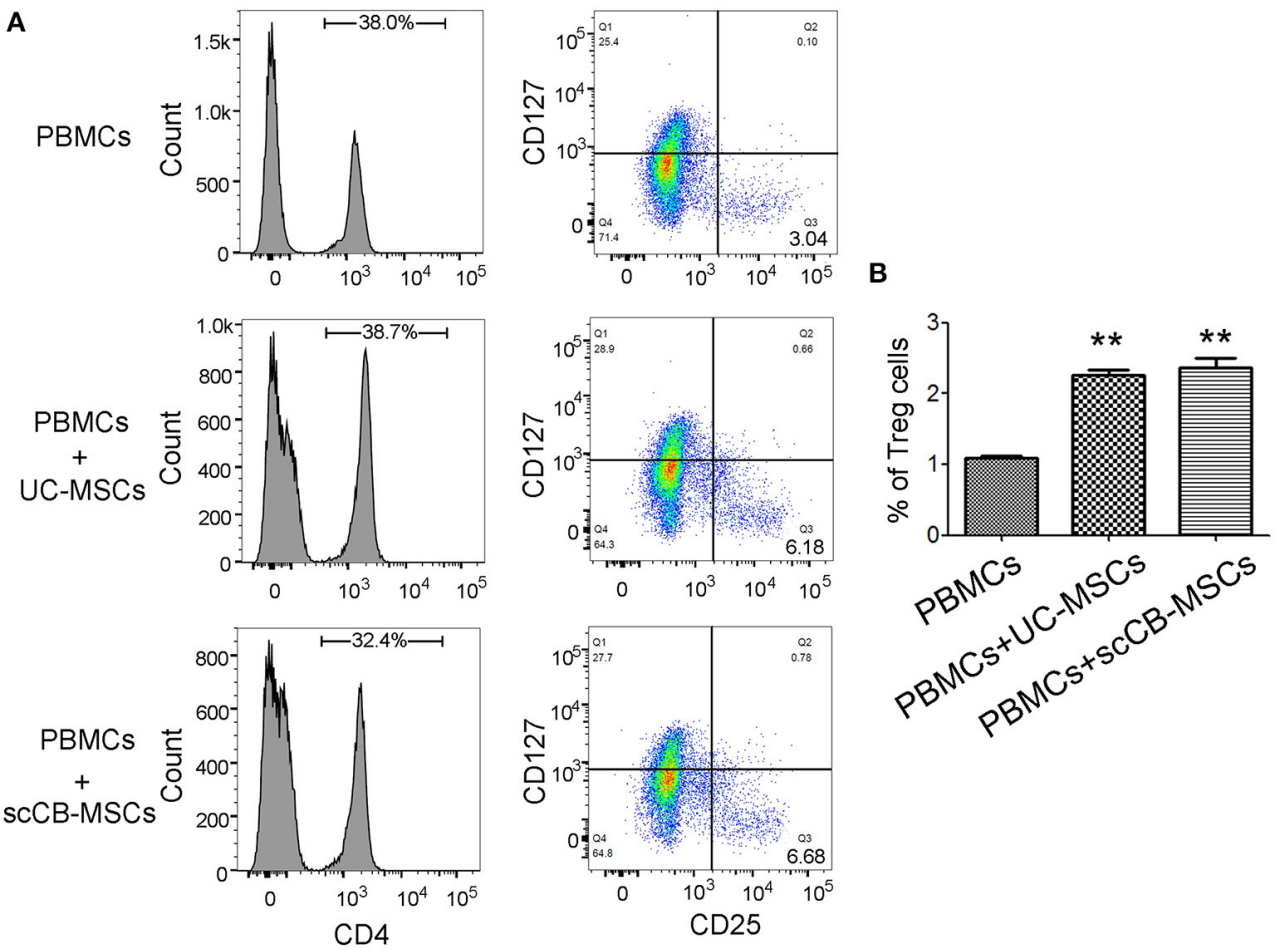
Inhibitory effects of scCB-MSCs on differentiation of lymphocytes into Th1 and Th17 cells. **(A)** Differentiating ability of PBMCs following PHA stimulation into T cell subsets was investigated in PBMCs alone and PBMCs co-cultured with UC-MSCs or scCB-MSCs. Cells under different conditions were allow to differentiate in the presence of PHA for 4 days, and then all cells were collected and permeabilized using 0.5% Triton X-100. After 10 min permeabilization, cells were stained with PE/PerCP/FITC/APC-conjugated antibodies targeting CD3, CD8, IFNγ, and IL-17, respectively. After staining, cells were analyzed using flow cytometry and cells displaying CD3^+^ and CD8^−^ (right bottom quadrant) were gated for further analysis of IFNγ and IL-17 expressions. **(B)** Percentages of Th1 (CD3^+^/CD8^+^/IFNγ; top bar graph) and Th17 (CD3^+^/CD8^+^/IL-17; bottom bar graph) cells under different conditions were summarized and all data represented as mean ± SD of three independent experiments. Asterisks represent significant difference (^**^P < 0.001) compared to the PBMCs alone group.

**Figure 6:**
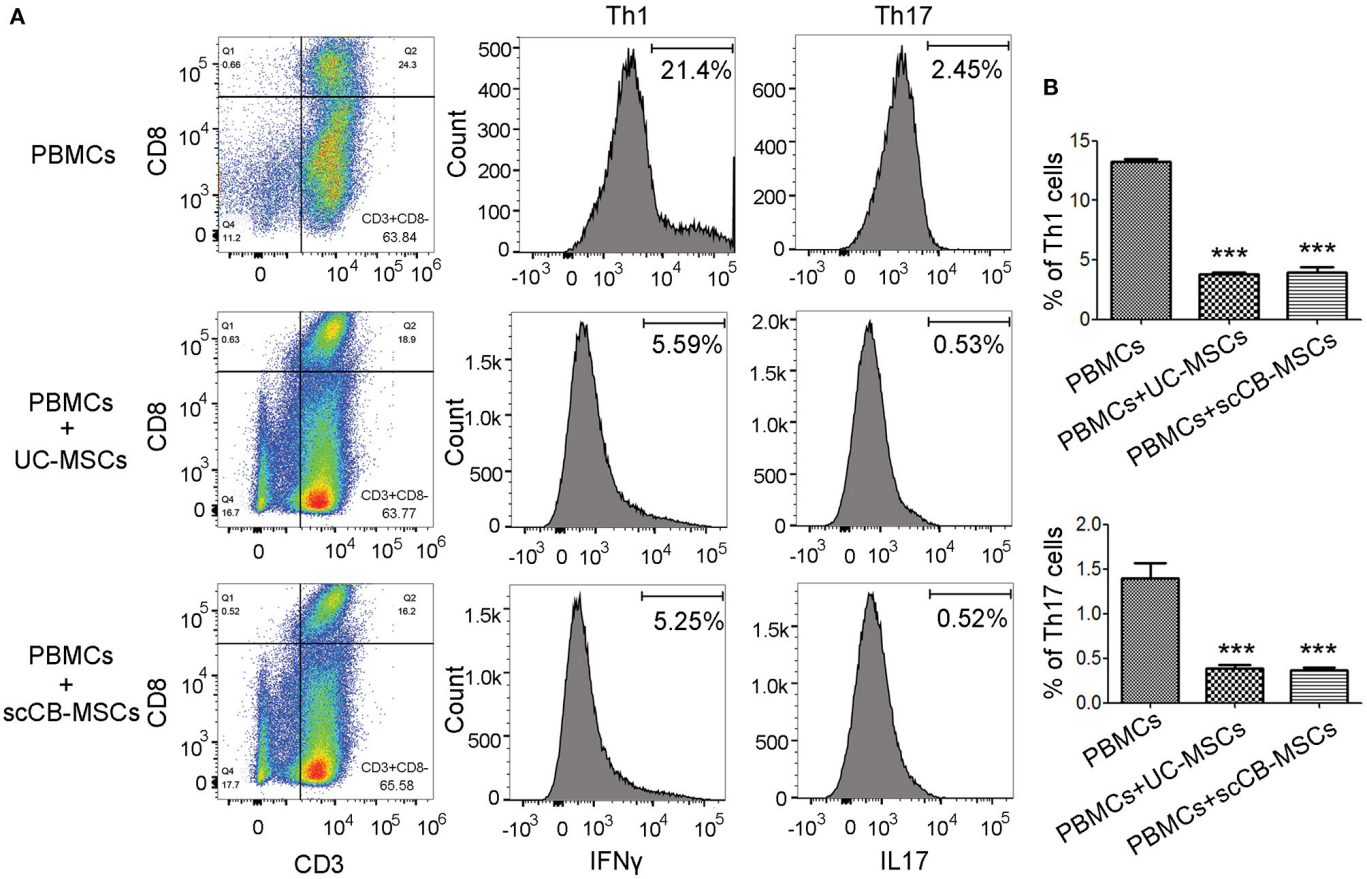
Co-culturing with scCB-MSCs promoting differentiation of lymphocytes into regulatory T cells. **(A)** Differentiating ability of PBMCs into T cell subsets was investigated in PBMCs alone and PBMCs co-cultured with UC-MSCs or scCB-MSCs for 4 days. Then all cells were collected and stained with FITC/PE/APC-conjugated antibodies targeting CD4, CD25, and CD127, respectively. After staining, cells were analyzed using flow cytometry and CD4^+^ cells were gated for de-fining regulatory T cells (i.e., CD25^+^ and CD127^−^; Q3). **(B)** Percentages of differentiating regulatory T cells (CD4^+^/CD25^+^/CD127^−^) under different conditions were summarized and all data represented as mean ± SD of three independent experiments. Asterisks represent significant difference (^***^P < 0.01) compared to the PBMCs alone group.

To further examine regulatory roles of scCB-MSCs in PBMCs differentiation, transwell-based co-culture system was employed. Following PHA stimulation, ~64% PBMCs displayed CD3+CD8– phenotype (i.e., T lymphocytes) in all groups. A further analysis of CD3^+^CD8^−^ cells on IFN-γ and IL-17 expression revealed that percentages of CD3^+^CD8^−^IFN-γ^+^ (i.e., Th1) and CD3^+^CD8^−^IL-17^+^ (i.e., Th17) were significantly de-creased compared to the group without co-culture. Specifically, ~21% of CD3^+^CD8^−^IFN-γ^+^ cells were observed in the PHA-stimulated group and this number significantly reduced to 5.59 and 5.25% when co-culturing with UC-MSCs and scCB-MSCs, respectively. Similarly, percentages of CD3^+^CD8^−^IL-17^+^ cells in the PHA-stimulated group also dropped from 2.45 to 0.53 and 0.52%, respectively (Figure 5). In comparison, percentage of cells differentiating into Treg in the presence of UC-MSCs and scCB-MSCs was also investigated. As shown in Figure 6A, ~35% of cells were CD4^+^ in PBMCs and further analysis of this population on CD25 and CD127 expression shown that the percentage of CD4^+^CD25^+^CD127^−^ roughly doubled from 3.04 to 6.18% (i.e., cells co-cultured with UC-MSCs) and 6.68% (i.e., cells co-cultured with scCB-MSCs). This represents a significant increase in Treg populations after scCB-MSCs treatment (Figure 6B).

## Discussion

MSCs were originally defined as colony-forming units-fibroblastic in the 1970s and since then have been extensively studied as an alternative source of stem cells for regenerative medicine (Friedenstein et al., 1970). Contrary to embryonic stem cells that entail the usage of early human embryos, MSCs are adult stem cells and thus, are less problematic in terms of ethical and legal considerations (Ding et al., 2011). This spurs numerous efforts to isolate and characterize MSCs from a variety of human tissues for their potential clinical applications (Pikuła et al., 2013; Miura, 2016; Van Pham et al., 2016; Yamada et al., 2019). Previous studies in our lab and others have demonstrated the neurogenic potential of CB-MSCs and their contributions to ameliorating neural injury in rat models (Abiko et al., 2018; Ma et al., 2019; Maeda et al., 2021). The overall goal of this study was to further investigate isolated CB-MSCs at clonal level with specific emphasis on the transdifferentiation capacity and immunoregulatory functions, which are two key features that need to be elucidated as required to move forward for the development of MSCs-based therapies.

Due to the variety in source tissues, isolation methods and culturing conditions, three minimal criteria must be met as considering isolated stem cells to be MSCs according to the International Society for Cellular Therapy (Dominici et al., 2006). In this study, isolated CB-MSCs exhibited fibroblast-like morphology under *in vitro* culture and capable of differentiating into adipocytes, osteoblasts and chondroblasts. Further phenotypic analysis revealed that isolated cells were negative for haematopoietic markers (i.e., CD14, CD34, and HLA-DR) and expressing CD73, CD90, and CD105. Collectively, these results further demonstrated that cells isolated from cranial bones are indeed MSCs with minimal haematopoietic stem cells contamination.

Isolated CB-MSCs represent a highly heterogenous pool of cells with differing propensity of differentiation into specific lineages. This prompts us to further investigate CB-MSCs at clonal level and indeed, although the expression of nestin (a neuroprogenitor marker) was comparable, scCB-MSCs exhibited more robust expression of Olig2, one marker of GABA progenitor (Goldberg and Coulter, 2014). As expect, scCB-MSCs were readily induced to differentiate into GABAergic neuron-like cells. We also found that cells from other colony exhibited MSC features (Supplementary Figure 3), but low levels in Olig2 expression (Supplementary Figure 4). These indicates that scCB-MSCs are committed to distinct types of neuron cells (i.e., glial cells and inhibitory interneuron) and further reinforce the fact that heterogeneity of MSCs should be considered and could potentially be utilized to improve differentiation efficiency into specific lineages for the treatment of specific diseases.

In addition to multilineage differentiation capacity, MSCs are capable of modulating activities of other immune cells and particularly, an immunosuppressive phenotype has been observed when MSCs were systematically or directly injected into injured sites (de Castro et al., 2019). This immunomodulatory feature of MSCs is critical to allow repairment of damaged tissues without robust activation of immune responses. In line with observations in immunoregulatory ability of UC-MSCs (Yang et al., 2020), scCB-MSCs also exhibited pronounced immunosuppressive phenotype that significantly inhibited immune cell proliferation and skewed T lymphocytes into regulatory phenotype. Although mechanistic details underlying observed immunosuppressive effects of CB-MSCs remain to be elucidated. Previous studies have suggested that paracrine activity are important in the immunomodulatory ability of MSCs (Gazdic et al., 2015; Yang et al., 2020). SEM analysis has seen CB-MSCs-derived extracellular vesicles (Ma et al., 2021). These small vesicles contain a range of bioactive molecules, such as proteins, fatty acids and nucleotides, and sever as an important mediator in intercellular communication (Raposo and Stahl, 2019). As a result, CB-MSCs likely exerted immunomodulatory effects *via* secretion of these vesicles.

In summary, we further investigated transdifferentiation capacity and immunomodulatory functions of CB-MSCs at clonal level. This represents the first report revealing the immunosuppressive phenotype and increased propensity of scCB-MSCs in comparison to CB-MSCs for GABAergic neural transdifferentiation. This study will serve as an important stepping-stone for understanding the *in vivo* roles of CB-MSCs and their potential applications in treating neurodegenerative diseases.

## Data Availability Statement

The original contributions presented in the study are included in the article/Supplementary Material, further inquiries can be directed to the corresponding authors.

## Ethics Statement

The studies involving human participants were reviewed and approved by Ethics Committee of Zhejiang Provincial People's Hospital (protocol code 2019KY15; October 10, 2019). The patients/participants provided their written informed consent to participate in this study.

## Author Contributions

KY, RL, RP, and JZ: conceptualization of the study and writing—review and editing. KY, RL, JL, and ZL: methodology. KY, RL, SF, QZ, YM, and GL: formal analysis. KY, RL, and QZ: investigation. KY: writing—original draft preparation. RP and JZ: supervision and funding acquisition. All authors have read and agreed to the published version of the manuscript.

## Funding

This project was supported by the General Project Funds from the Health Department of Zhejiang Province (Grant No: 2022KY584); the General Project Funds from the Education Department of Zhejiang Province (Grant No: Y202146121); Zhejiang Province Public Welfare Technology Application Research Project (Grant No: LGF21H090019).

## Conflict of Interest

QZ and RP were employed by S-Evans Biosciences. The remaining authors declare that the research was conducted in the absence of any commercial or financial relationships that could be construed as a potential conflict of interest.

## Publisher's Note

All claims expressed in this article are solely those of the authors and do not necessarily represent those of their affiliated organizations, or those of the publisher, the editors and the reviewers. Any product that may be evaluated in this article, or claim that may be made by its manufacturer, is not guaranteed or endorsed by the publisher.

## Supplementary Material

Supplementary Figure 1 Differentiation of CB-MSCs into classical mesenchymal derivatives were validated by real-time quantitative polymerase chain reaction. mRNA expressions of *Bglap, Leptin (LP)*, and *Collagen II*, which represented differentiation into osteoblasts, adipocytes and chondrocytes respectively, were detected. GAPDH was used as an internal control.

Supplementary Figure 2 mRNA expressions of Nestin and Olig2 were detected by real-time quantitative polymerase chain reaction. GAPDH was used as an internal control. ^**^*P* < 0.01.

Supplementary Figure 3 Morphological and phenotypic characterizations of another colony derived CB-MSCs. **(A)** Morphology of cells following *in vitro* culture at passage 5 was imaged using phase contrast microphotography. **(B)** Mineral deposition, Adipogenic and chondrogenic differentiation was observed after 3 weeks of *in vitro* differentiation using Alizarin Red, Oil Red O and Alcian Blue staining, respectively. Scar bar = 50 μm. **(C)** Phenotypic analysis of surface markers using flow cytometry. Cells were stained with FITC/PE-conjugated primary antibodies targeting selected surface markers (i.e., CD73, CD90, CD105, CD14, CD34, and HLA-DR). As controls, MSCs were also stained with fluorochrome-conjugated isotype IgGs to identify positive cells.

Supplementary Figure 4 Detection of olig2 expression of another colony derived CB-MSCs by Immunofluorescence staining. Cells were labeled with Olig2 antibody (green) and counterstained with DAPI (blue). Scale Bars = 50 μm.

Supplementary Table 1 Primers and Annealing temperatures.
