# Supplementary figures and images for: Phenotypic and Functional Characterizations of Mesenchymal Stem/Stromal Cells Isolated From Human Cranial Bone Marrow
Source: Front Neurosci. 2022 Jun 7;16:909256. doi: 10.3389/fnins.2022.909256 (PMC9209782; doi:10.3389/fnins.2022.909256)

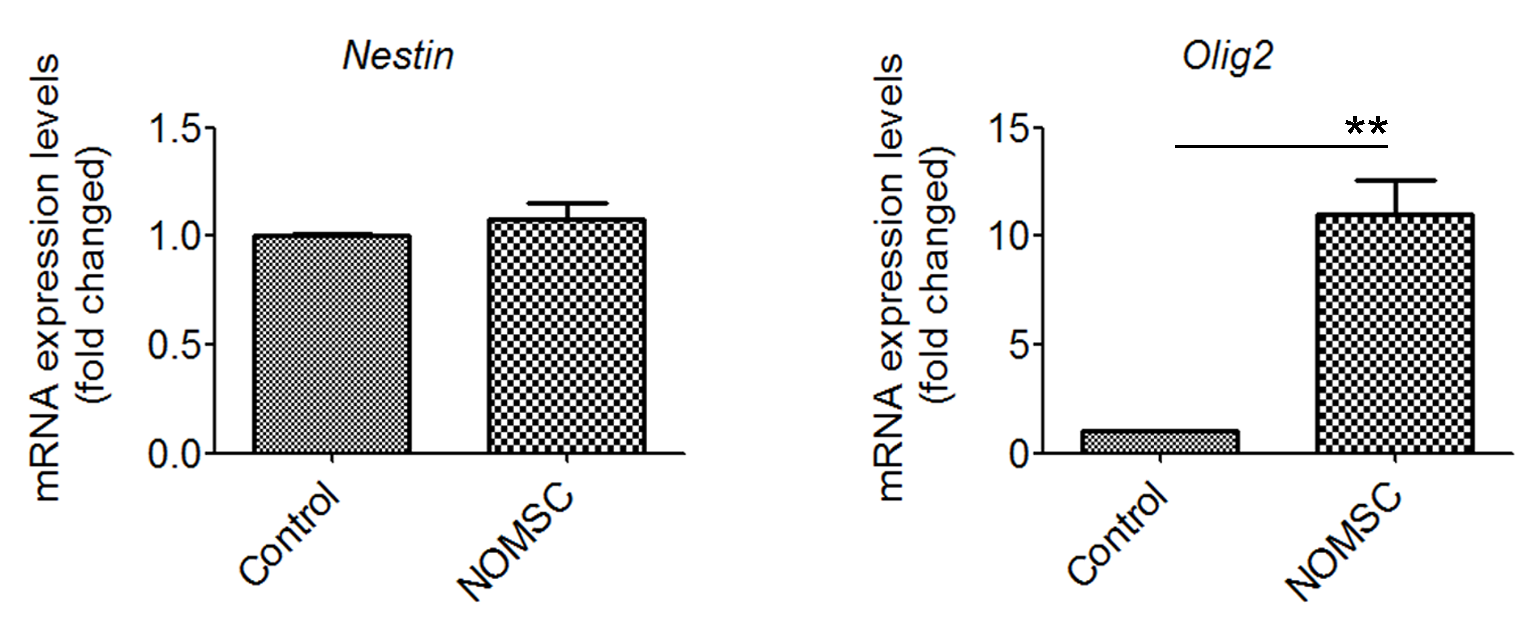

Supplement: Supplementary Figure 1 — Differentiation of CB-MSCs into classical mesenchymal derivatives were validated by real-time quantitative polymerase chain reaction. mRNA expressions of Bglap, Leptin (LP), and Collagen II, which represented differentiation into osteoblasts, adipocytes and chondrocytes respectively, were detected. GAPDH was used as an internal control. [file Image_1.TIF]

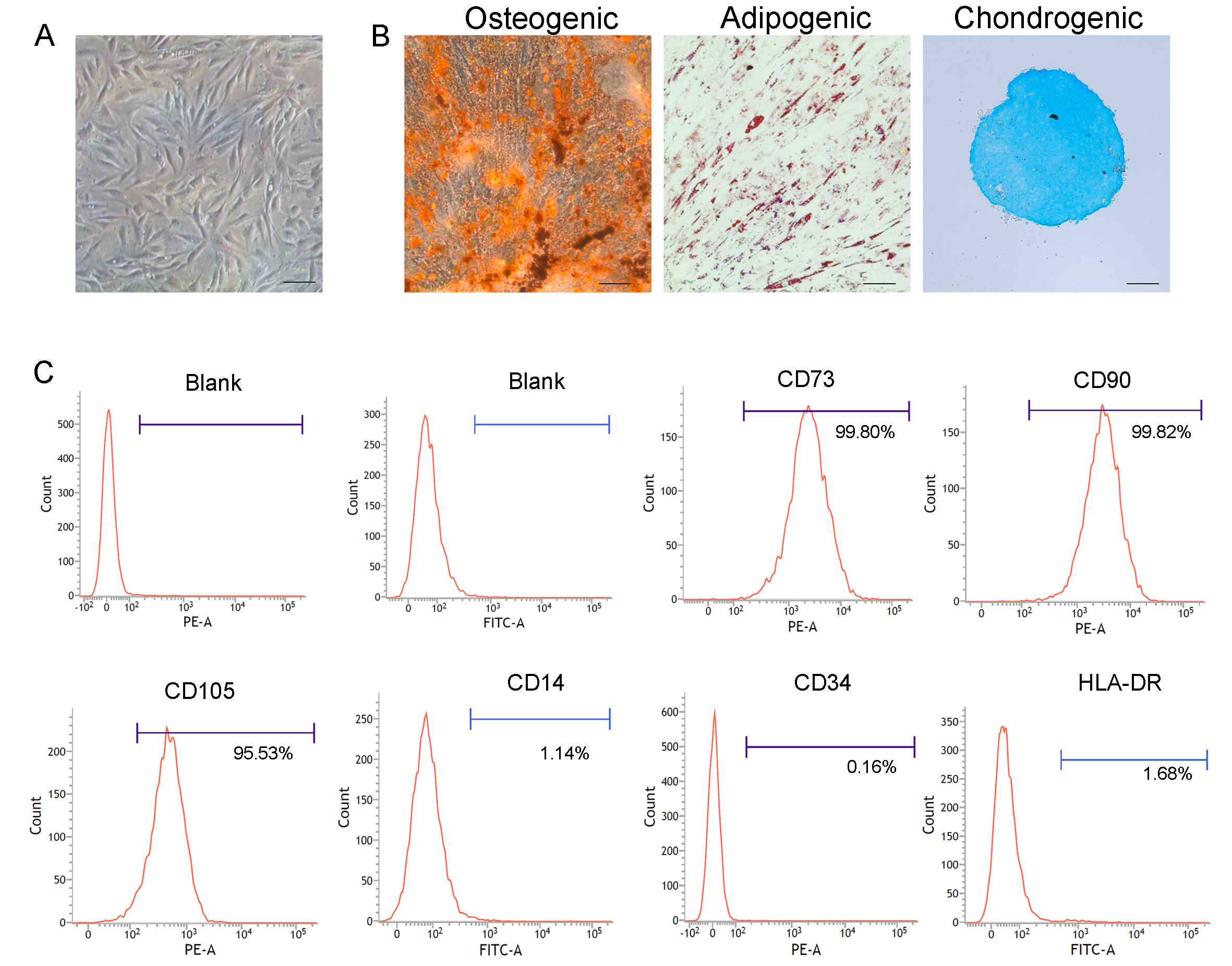

Supplement: Supplementary Figure 2 — mRNA expressions of Nestin and Olig2 were detected by real-time quantitative polymerase chain reaction. GAPDH was used as an internal control. **P < 0.01. [file Image_2.TIF]

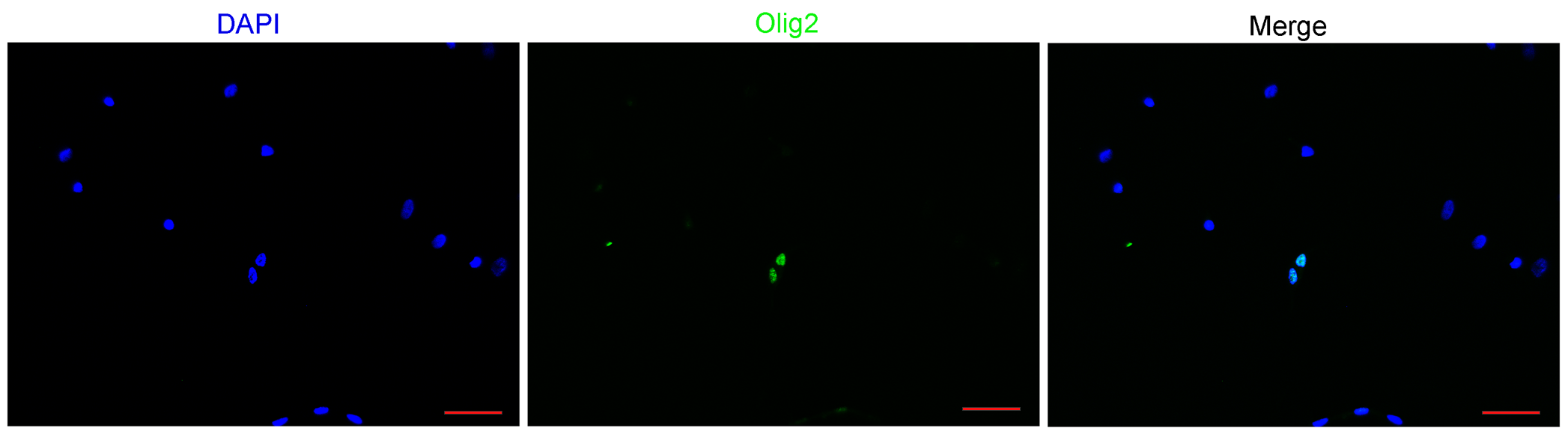

Supplement: Supplementary Figure 3 — Morphological and phenotypic characterizations of another colony derived CB-MSCs. (A) Morphology of cells following in vitro culture at passage 5 was imaged using phase contrast microphotography. (B) Mineral deposition, Adipogenic and chondrogenic differentiation was observed after 3 weeks of in vitro differentiation using Alizarin Red, Oil Red O and Alcian Blue staining, respectively. Scar bar = 50 μm. (C) Phenotypic analysis of surface markers using flow cytometry. Cells were stained with FITC/PE-conjugated primary antibodies targeting selected surface markers (i.e., CD73, CD90, CD105, CD14, CD34, and HLA-DR). As controls, MSCs were also stained with fluorochrome-conjugated isotype IgGs to identify positive cells. [file Image_3.TIF]

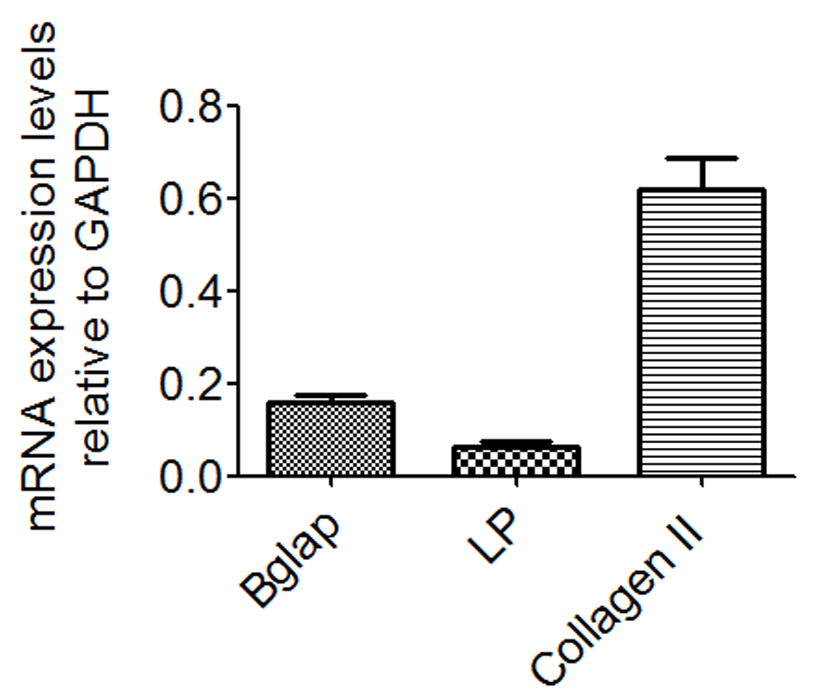

Supplement: Supplementary Figure 4 — Detection of olig2 expression of another colony derived CB-MSCs by Immunofluorescence staining. Cells were labeled with Olig2 antibody (green) and counterstained with DAPI (blue). Scale Bars = 50 μm. [file Image_4.TIF]
