# Supplementary material for: Phenotypic and Functional Characterizations of Mesenchymal Stem/Stromal Cells Isolated From Human Cranial Bone Marrow
Source: Front Neurosci. 2022 Jun 7;16:909256. doi: 10.3389/fnins.2022.909256 (PMC9209782; doi:10.3389/fnins.2022.909256)
Supplement: Supplementary Table 1 — Primers and Annealing temperatures. [file Table_1.DOCX]

# Supplementary Materials and Methods

**Reverse transcription and quantitative polymerase chain reaction (RT-qPCR)**

RNA from cells was extracted using TRIzol reagent (Life Technologies, Carlsbad, CA), and the amount was quantified using a NanoDrop spectrophotometer (Thermo Fisher Scientific, Waltham, MA). cDNA synthesis was performed using reverse transcriptase (Promega, Madison, WI) and random hexamer primers (Promega) following the manufacturer’s instructions. The cDNA product was then used for real-time PCR using a SYBR Green Master Mix (Takara, Dalian, China). Gene expression levels were normalized against the glyceraldehyde-3-phosphate dehydrogenase (GAPDH) housekeeping gene expression level. Relative expression levels were obtained using the ΔCt method. Amplifications were performed in triplicate on three biological replicates. Specific primers are listed in Table 1.

**Table 1: Primers and Annealing temperatures**

| Gene | Sequence (5’-3’) | Annealing temperature |
| --- | --- | --- |
| Bglap-F | CACTCCTCGCCCTATTGGC | 62°C |
| Bglap -R | CCCTCCTGCTTGGACACAAAG |  |
| Leptin-F | TCCAAGATGACACCAAAACCC | 62°C |
| Leptin-R | AAGGCCAGCACGTGAAGAAGA |  |
| Collagen II-F  Collagen II-R | CCCCAGGTGAAGGTGGAAAAC | 62°C |
|  | GACCATCAGTGCCAGGAGTGC |  |
| Nestin-F | CTCACCCTTGCCTGCTACCCT | 63°C |
| Nestin-R | TGCCCTCTATGGCTGTTTCTTTCT |  |
| Olig2-F | ATGCACGACCTCAACATCGCCA | 60°C |
| Olig2-R | ACCAGTCGCTTCATCTCCTCCA |  |
| GAPDH-F | GGAGCGAGATCCCTCCAAAAT | 62°C |
| GAPDH-R | GGCTGTTGTCATACTTCTCATGG |  |
